# SequenceCEROSENE: a computational method and web server to visualize spatial residue neighborhoods at the sequence level

**DOI:** 10.1186/s13040-016-0083-7

**Published:** 2016-01-27

**Authors:** Florian Heinke, Sebastian Bittrich, Florian Kaiser, Dirk Labudde

**Affiliations:** Department of Applied Computer and Biosciences, University of Applied Sciences Mittweida, Technikumplatz 17, Mittweida, 09648 Germany

**Keywords:** Molecular structure, Visualization, Sequence, Residue neighborhood, Color encoding

## Abstract

**Background:**

To understand the molecular function of biopolymers, studying their structural characteristics is of central importance. Graphics programs are often utilized to conceive these properties, but with the increasing number of available structures in databases or structure models produced by automated modeling frameworks this process requires assistance from tools that allow automated structure visualization. In this paper a web server and its underlying method for generating graphical sequence representations of molecular structures is presented.

**Results:**

The method, called SequenceCEROSENE (color encoding of residues obtained by spatial neighborhood embedding), retrieves the sequence of each amino acid or nucleotide chain in a given structure and produces a color coding for each residue based on three-dimensional structure information. From this, color-highlighted

sequences are obtained, where residue coloring represent three-dimensional residue locations in the structure. This color encoding thus provides a one-dimensional representation, from which spatial interactions, proximity and relations between residues or entire chains can be deduced quickly and solely from color similarity. Furthermore, additional heteroatoms and chemical compounds bound to the structure, like ligands or coenzymes, are processed and reported as well.

To provide free access to SequenceCEROSENE, a web server has been implemented that allows generating color codings for structures deposited in the Protein Data Bank or structure models uploaded by the user. Besides retrieving visualizations in popular graphic formats, underlying raw data can be downloaded as well. In addition, the server provides user interactivity with generated visualizations and the three-dimensional structure in question.

**Conclusions:**

Color encoded sequences generated by SequenceCEROSENE can aid to quickly perceive the general characteristics of a structure of interest (or entire sets of complexes), thus supporting the researcher in the initial phase of structure-based studies. In this respect, the web server can be a valuable tool, as users are allowed to process multiple structures, quickly switch between results, and interact with generated visualizations in an intuitive manner.

The SequenceCEROSENE web server is available at https://biosciences.hs-mittweida.de/seqcerosene.

## Background

To understand the role governed by a protein or biopolymer of interest, the study of its structure is often of great importance. In the process of investigation, schematic representations of interactions between structural elements provide simplified but informative visualizations that help the researcher to understand and illustrate but also to communicate essential molecular characteristics [1]. Such characteristics range from the general topological arrangement of entire domains and secondary structure elements to the level of residue interaction networks.

One can distinguish between two categories of representations. The first category includes schematics which we here refer to as 2.5-dimensional representations. These diagrammatic representations illustrate spatial neighborhood and arrangements of structural elements. Given atomic coordinate data as input, locations of the elements in two-dimensional space are computed that resemble the three-dimensional topology as closely as possible. For example, TopDraw [2], HERA [1], Pro-origami [3] and TOPS [4] are tools for generating such representations. Furthermore, precomputed topology diagrams of solved structures can be found at the PDBsum database [5]. PROTTER [6] is a recent addition to this class of tools and is specifically tailored for visualizing *α*-helical membrane protein topology. In contrast to aforementioned tools, PROTTER derives topology information only from predictions made from sequence, whereas available structure information is not considered. On the level of residue interactions, intuitive and clear representations are restricted to subsets of residues and their associated interactions, due to the vast amount of interactions present in a structure. Approaches such as RING [7] propose 2.5-dimensional graph-based representations of residue/interaction sets. In this respect, the Protein Graph Repository provides access to nearly 190,000 graphs generated from about 94,000 protein structures [8].

Although diagrammatic representations feature visual clarity, the process of low-dimensional mapping achieved by reducing dimensionality yields a drawback not obvious to the user: dimensionality reduction is generally accompanied by information loss and unavoidable morphing effects, both depending on structural complexity. Eventually, information loss and morphing effects reduce mapping quality. Thus, resemblance of structure topology between the actual three-dimensional structure and its corresponding 2.5-dimensional visualization is not necessarily of the same quality for any given protein structure.

The second category of techniques avoid this problem by employing bijective projections. Here, color maps illustrating residue-residue adjacencies or distances are straightforward visualizations and can be produced using a number of available tools, such as CMView [9] and CMA [10]. However, compared to 2.5-dimensional representations such maps cannot achieve the same degree of clarity, which makes them difficult to interpret intuitively. Thus, these visualizations are of greater use if interactivity with 3D structure viewers is implemented (such as realized by CMView).

In this paper, an effective and straightforward approach for generating intuitive representations of spatial residue neighborhood is proposed, which we refer to as SequenceCEROSENE (color encoding of residues obtained by spatial neighborhood embedding). SequenceCEROSENE produces color-highlighted sequences of amino acid, DNA, and RNA chains present in the query structure, including bound chemical compounds and heteroatoms. Using a straightforward transformation of the atomic coordinates into RGB color space, color-highlighting of individual residues and compounds corresponds to their location in the three-dimensional space. Thus, similarity of color present in color encoded sequences between residues or compounds illustrates spatial proximity. The overall presentation of the visualization resembles a colorized set of FASTA formatted sequences, where bound compounds/heteroatoms are reported individually. SequenceCEROSENE aims at producing representations containing as much structural information as possible while allowing intuitive interpretation. In fact, by decoding the RGB content of individual residues, the three-dimensional structure of the complex can be well approximated.

In the following sections, the SequenceCEROSENE method is introduced and demonstrated, followed by a brief presentation of the web server.

## Method and discussion

Using a bijective projection of structural data, SequenceCEROSENE produces visualizations that can be assigned to the second representation category. In the first step of computation, atomic coordinates representing residues, compounds and heteroatoms are determined. In this step, *C*_*β*_ atoms, *C*_*α*_ atoms, side chain centroids, or terminal heavy side chain atoms can be considered. The set *P* of representative coordinates is then transformed into the three-dimensional RGB color space. In the first step of the transformation, the structure centroid *p*_*c*_ is computed as follows:

(1) $$ p_{c} = \frac{1}{|P|}\sum_{p_{i} \in P} p_{i}.  $$

In the next step, the maximum span *s* for each of the three coordinates *x*, *y* and *z* is computed. Equation 2 illustrates the computation for the *x* coordinate.

(2) $$ \mathrm{s}_{x} = \mathop{\arg\max}_{p_{i} \in P} {|p_{i,x} - p_{c,x}|}   $$

The maximum coordinate span *s*_*max*_ corresponds to the maximum of *s*_*x*_, *s*_*y*_ and *s*_*z*_. Finally, *s*_*max*_ and *p*_*c*_ are used to normalize, transform and translate the coordinates *P* to corresponding RGB coordinates *C*:

(3) $$\begin{array}{@{}rcl@{}} c_{i,red} &=& 128 * \frac{p_{i,x} - p_{c,x}}{s_{max}} + 128  \\ c_{i,green} &=& 128 * \frac{p_{i,y} - p_{c,y}}{s_{max}} + 128  \\ c_{i,blue} &=& 128 * \frac{p_{i,z} - p_{c,z}}{s_{max}} + 128. \end{array} $$

**Fig. 1:**
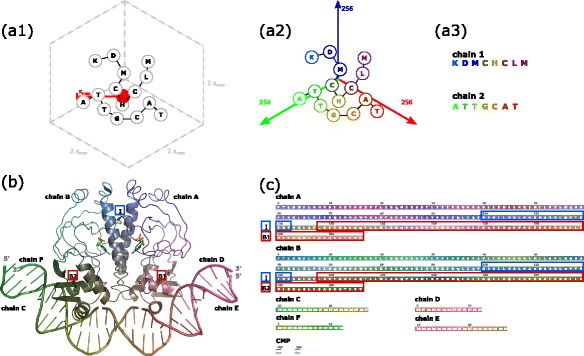
Schematic workflow of SequenceCEROSENE and example output shown for transcription promoter CAP (catabolite activator protein, PDB-Id 1cgp [12]). Upon computing the maximum spatial span *s*_*max*_, the given molecular complex is embedded in a cubic bounding box (**a1**) and, based on this, translated into RGB color space (**a2**). Eventually, corresponding sequences are color-coded in accordance to three-dimensional locations of residues (**a3**) from which structural residue neighborhood can be quickly deduced. For example, interfacial residues between both peptide chains in CAP are located in a region of 22 residues relating to a helix-helix interaction (highlighted by blue box I in (**b**) and (**c**)). These residues can intuitively be identified by their common grey-blue coloring in the color encoded sequences. Further, color encoding intuitively reflects that the two dsDNA molecules are comprised by chains C and F as well as D and E. Here, note the reversed color coding of the bound chains, indicating anti-parallel binding. In addition, the DNA binding domain in both peptide chains corresponds to the C-terminal region highlighted by red box B1 respectively B2. Color encoding of the sequences shows that dsDNA D-E and C-F are bound to regions B1 and B2, respectively. Note that in (**b**) representations of secondary structure elements outside of regions I, B1 and B2 are neglected for visual clarity

The process can also be interpreted as defining a cubic box surrounding the structure, followed by rescaling both the box and the representative coordinates *P* to match a cube side length of 256 (see Fig. 1a for an illustration). Here, *s*_*max*_ determines the initial relative size of the box prior to rescaling. However, for small and compact structures small relative box sizes are obtained (as in case of mini-proteins or small nucleic acid structures), which results in representations that overstate actual small sizes by reporting large spreads in the RGB space. To avoid this contradiction, a minimum value for *s*_*max*_ has to be considered if the maximum coordinate span obtained from structure is observed to be smaller. In SequenceCEROSENE a minimum *s*_*max*_ of 23 Å is employed. This value has been chosen by analyzing the *s*_*max*_ distribution of 1,172 structures with less than 40 residues extracted from the Protein Data Bank (PDB, [11]). The 95 % percentile of this distribution corresponds to 23 Å and has been determined to define minimum *s*_*max*_.

Finally, SequenceCEROSENE provides color-encoded illustrations of corresponding amino acid and nucleotide sequences. Bound compounds and heteroatoms are visualized separately. This representation is both intuitive and powerful, since residue neighborhood can be directly perceived by color similarity and the structural information between representative coordinates is maintained. In theory, RGB color contents for each residue in the generated representation can be decoded in order to restore the actual three-dimensional coordinates. Information loss only occurs during the representative coordinate determination process, as centroid coordinates and single atoms are considered.

As an example, the structure of transcription promoter CAP (catabolite activator protein, PDB-Id: 1cgp [12]) and the corresponding sequence representation generated by SequenceCEROSENE are shown in Fig. 1b and c, respectively.

## Implementation

A web server has been implemented to provide free access to SequenceCEROSENE for the scientific community. Upon entering a valid Protein Data Bank (PDB) identifier [11] or uploading single or multiple structures in PDB format, the server computes color encodings and presents these to the user interactively. As discussed in the Method and discussion section, the set of residue representative coordinates needs to be determined prior to processing. By default, *C*_*β*_ atoms (*C*_*α*_ atoms for glycines) are selected for amino acids. However, as an advanced option, the user can also choose between using *C*_*α*_ atoms, terminal heavy side chain atoms, or side chain centroid coordinates. For nucleotide residues and compounds, the centroid coordinate is always considered.

Furthermore, multiple structures can be processed in one submission. Therefore, the user has to provide an archive containing the PDB files in question. All commonly used archive file formats are supported. If a PDB-Id is provided, the web server retrieves the data from a local weekly-updated snapshot of the PDB. If no local PDB file for a user query is present, a fallback option is implemented to automatically retrieve the data from the PDB using RESTful web services.

**Fig. 2:**
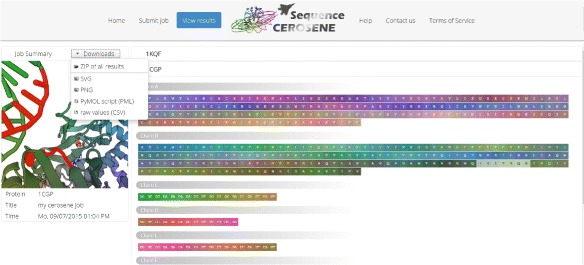
SequenceCEROSENE server result page for CAP. Besides presenting visualizations and providing links for downloading generated color encodings, the web servers allows to interact with generated representations. As shown, residues can be highlighted in sequence and structure by either making a selection in the sequence or in the structure as both parts are interconnected. The protein structure viewer is realized using PV [15]. Furthermore, multiple structures can be processed in one submission by uploading an archive file in any popular format. In this example, the structure of *E. coli* formate dehydrogenase N (PDB-Id: 1kqf) has been uploaded in addition to CAP. Result pages are organized in individually selectable tabs, thus allowing to quickly switch between processed structures. In the case shown here, the results for 1kqf are also available via an additional tab

After successful query processing, the result page is presented, where the user interface allows interacting with generated visualizations and structures. If multiple structures have been submitted, the user can skip between generated outputs using corresponding tabs. Links are provided for downloading generated visualizations as PNG and SVG graphics. If desired, users can also retrieve underlying raw data in text format. To further inspect the obtained color embedding of the three-dimensional structure locally, a PyMOL PML file [13] is generated. An exemplary result page is shown in Fig. 2. Here, to accompany the example illustrated in the Method and discussion section, the structure of CAP has been used as input.

## Conclusion

The utilization of dedicated 3D structure visualization programs is the method of choice for studying and understanding general spatial characteristics of biopolymer structural data. Techniques for generating "condensed" visualizations portraying such characteristics are widely used in the process, as they support the researcher in constructing a "mental image" of the structure in question.

Generated visualizations can be classified into two general categories: 2.5-dimensional diagrammatic representations of protein topology (the general spatial arrangements of structural elements) and visualizations on residue-residue interaction level (such as residue distance or interaction matrices). As discussed, both visualization categories are limited by trade-offs between visual clarity and amount of information presented.

SequenceCEROSENE aims at filling the gap between techniques of both categories by providing an intuitive visualization on sequence level while keeping information loss minimal. Furthermore, in contrast to most methods, structure data of protein, DNA, RNA and complexes thereof can be processed, making this technique applicable to a variety of biopolymers.

Analogous to available automated structure visualization techniques, the main intention of the method is to provide visual guidance to researchers and students in the initial phase of studying structural data, especially if the number of structures is large. The implemented web server allows processing, downloading, and interacting with generated visualizations, however future implementations of SequenceCEROSENE as plug-ins or add-ins for common visualization programs, such as PyMOL [13] and VMD [14], could improve accessibility. Considering the simplicity of the methodological idea, we hope that researchers are inspired to adapt this technique, thus giving rise to implementations that are specifically tailored toward their own research and analysis pipelines.

## Availability and Requirements

The server can be accessed using common web browsers. For displaying protein structures, PV requires WebGL to be supported, which, however, is the case for the majority of available browsers. To enable these WebGL-based features for unsupported browsers, manual installation of browser-specific WebGL add-ons and libraries is required. No further requirements are necessary. The Sequence- CEROSENE web server is freely available to the scientific community.
